# MEG3 Promotes Differentiation of Porcine Satellite Cells by Sponging miR-423-5p to Relieve Inhibiting Effect on SRF

**DOI:** 10.3390/cells9020449

**Published:** 2020-02-15

**Authors:** Xiaofang Cheng, Long Li, Gaoli Shi, Lin Chen, Chengchi Fang, Mengxun Li, Changchun Li

**Affiliations:** 1 A Key Laboratory of Agricultural Animal Genetics, Breeding, and Reproduction of the Ministry of Education and Key Laboratory of Swine Genetics and Breeding of the Ministry of Agriculture, Huazhong Agricultural University, Wuhan 430070, China; xfchengsunny@163.com (X.C.); 15207165483@163.com (L.L.); shigaoli@webmail.hzau.edu.cn (G.S.); 18848970716@163.com (L.C.); fangchengchi22@aliyun.com (C.F.);; 2 The Cooperative Innovation Center for Sustainable Pig Production, Huazhong Agricultural University, Wuhan 430070, China

**Keywords:** porcine satellite cells, myoblast differentiation, *MEG3*, ceRNA, miR-423-5p

## Abstract

Although thousands of long noncoding RNAs (lncRNAs) have been identified in porcine growth and development, the regulation mechanisms of functional lncRNAs have not been well explored. In this study, using 5′- and 3′-rapid amplification of cDNA ends (RACE) assays, we obtained two different variants of lncRNA maternally expressed gene 3 (*MEG3*), namely, *MEG3* v1 and *MEG3* v2, that were both highly expressed in porcine skeletal muscle and in the early stage of the differentiation of porcine satellite cells. Moreover, we identified the core transcript *MEG3* v2. Functional analyses showed that *MEG3* overexpression could effectively arrest myoblasts in the G1 phase, inhibit DNA replication, and promote myoblast differentiation, whereas *MEG3* knockdown resulted in the opposite effects. Interestingly, the expression of serum response factor (*SRF*), a crucial transcription factor for myogenesis process, remarkably increased and decreased in mRNA and protein levels with the respective overexpression and knockdown of *MEG3*. Dual luciferase reporter assay showed that *MEG3* could attenuate the decrease of luciferase activity of *SRF* induced by miR-423-5p in a dose-dependent manner. *MEG3* overexpression could relieve the inhibitory effect on *SRF* and myoblast differentiation induced by miR-423-5p. In addition, results of RNA immunoprecipitation analysis suggested that *MEG3* could act as a ceRNA for miR-423-5p. Our findings initially established a novel connection among *MEG3*, miR-423-5p, and *SRF* in porcine satellite cell differentiation. This novel role of *MEG3* may shed new light on understanding of molecular regulation of lncRNA in porcine myogenesis.

## 1. Introduction

In mammalian genomes, only 2% of transcripts are translated into proteins. The vast majority of transcripts are noncoding RNAs, including microRNAs (miRNAs), Piwi-interacting RNAs, circular RNAs, and long noncoding RNAs (lncRNAs) [1]. Recently, an increasing number of researchers have focused on lncRNAs, which are a type of RNA with lengths of more than 200 nt and lack protein-coding potential [2]. They are characterized by less abundant, less evolutionarily conserved, and spatio-temporal specific expression profiles [3,4,5]. Thousands of functional lncRNAs have been identified to be involved in multiple biological processes, such as X chromosome inactivation, genomic imprinting, stem cell maintenance, embryonic development, myogenesis, immunity, and tumorigenesis [6,7,8,9,10,11].

Mammalian skeletal muscle development is a complex process, which includes the following phases: somite commitment into progenitors, myoblast proliferation, migration, fusion, and final adaptation into fast-twitch and slow-twitch muscle fibers [12]. These development phases are regulated by the cascade control of multiple transcription factors, including myogenic regulatory factor 4 (*MRF4*), myogenic differentiation (*MyoD*), muscle *bHLH* proteins, *Myf5*, myogenin (*MyoG*), and myocyte enhancer factor 2 (MEF2) family [13,14,15]. A small number of lncRNAs, such as *lncRNA Dum* [16], *MUNC* [17,18], *Linc-MD1* [19], *Lnc-mg* [20,21], *LncMyoD* [22], *Linc-RAM* [23], *Linc-YY1* [24], *SYISL* [25], and *lncRNA Irm* [26], have been characterized to mediate biological processes of myoblasts during skeletal muscle development. These lncRNAs regulate skeletal muscle myogenesis and regeneration through various mechanisms, including chromosome modification, transcription activation, molecular sponge activity, competitive binding, mRNA translation, and protein stability.

The lncRNA maternally expressed gene 3 (*MEG3*) on chromosome 7 is a conserved lncRNA among pig, humans, mice, and cow [22,27,28]. *MEG3* is abundantly expressed in the paraxial mesoderm and is involved in callipyge phenotype of sheep, causing significant changes in muscle development and low fat content in the waist and hindquarters [29,30,31,32,33]. The knockout of *MEG3* leads to skeletal muscle developmental defects and perinatal death [34]. In Qinchuan cattle, lncRNA *MEG3* serves as a molecular sponge of miR-135, attenuating the suppressive effects of miR-135 upon *MEF2C* and thereby promoting skeletal differentiation [35]. Wang et al. proposed the potential roles of the MEF2A–MEG3/DIO3–PP2A signaling regulatory axis in bovine myoblast differentiation [36]. Downregulation of *MEG3* can promote the proliferation and migration of smooth muscle cells of human pulmonary arteries [37]. In pigs, *MEG3* has high expression levels in prenatal and early postnatal skeletal muscle [38], and four single nucleotide polymorphisms of *MEG3* identified from Large White pigs are associated with meat-producing traits [39]. These findings indicated that *MEG3* may be involved in myogenesis and contribute to skeletal muscle development in pigs. However, the molecular mechanisms need to be further explored.

To verify our speculation, we further detected the expression profile of *MEG3* and explored its function and molecule mechanism in the myogenesis of porcine satellite cells. In this study, we observed that the expression level of *MEG3* in skeletal muscle was higher than that in other tissues, and *MEG3* was differentially expressed in the myogenesis of porcine satellite cells. Moreover, *MEG3* could effectively inhibit myoblast proliferation and promote myoblast differentiation. Mechanistically, *MEG3*, as a competing endogenous RNA (ceRNA), promotes the differentiation of porcine satellite cells by sponging miR-423-5p to relieve the inhibiting effect on serum response factor (*SRF*). Our results may contribute to a better understanding of the lncRNA–miRNA–target-gene regulatory network in the differentiation of porcine satellite cells.

## 2. Materials and Methods

### 2.1. Animals

Animals used in this study were <7-days-old male Large White piglets. Six piglets were euthanized, and organs and tissues were collected after dissection for tissue expression profile analysis. For porcine satellite cells isolation, the hind leg muscles from three other piglets were rapidly pooled, minced and digested. Animals care and experimentation in this study have been performed in accordance with the National Research Council Guide for the Care and Use of Laboratory Animals and have been approved by the Institutional Animal Care and Use Committee of Huazhong Agricultural University, Wuhan, China (permit HZAUSW2015-0003).

### 2.2. Cell Culture

Satellite cells were primarily isolated from hind leg muscles of <1-week-old male piglets. Briefly, skeletal muscles were minced into pieces and digested with 300 U/mL type II collagenase (Gibco; Gaithersburg, MD, USA) in a shaking water bath at 37 °C for 2.5 h. After having been terminated with high-glucose Dulbecco’s modified Eagle’s medium (DMEM; Gibco) supplemented with 10% fetal bovine serum (FBS; Gibco), the cell suspension was filtered through 100, 70, and 40 μm filters to remove tissue debris. Afterwards, the cell pellet was ultimately resuspended and cultured in RPMI-1640 medium supplemented with 20% FBS (Gibco), 0.5% Chicken Embryo Extract (Gemini, Woodland, CA, USA), 1% GlutaMax (Gibco), 1% NEAA (Gibco), 1% Anti-Anti (Gibco), 2.5 μg/L basic fibroblast growth factor (bFGF; Invitrogen, Grand Island, NY, USA). Due to differential adhesion property, fibroblasts among the mixed cells were removed after being incubated in uncoated plates for 2.5 h. The purified satellite cells were transferred into the Matrigel (BD Biosciences, CA, USA) coated plates for proliferation cultures. When porcine satellite cells were grown to 90% confluence, they were transferred into DMEM supplemented with 5% Horse Serum (HS; Gibco) to induce differentiation.

The PK15 cells were cultured in DMEM supplemented with 10% FBS (Gibco).

All cells described above were incubated at 37 °C in 5% CO_2_.

### 2.3. RNA Oligonucleotide and Plasmid Construction

The miR-423-5p mimic, mimic negative control (NC) and the antisense oligonucleotide (ASO) oligo against *MEG3* and scrambled oligo were purchased from RiboBio (Guangzhou, China). Small interfering RNA (siRNA) of *SRF* and scrambled oligo were designed and synthesized from GenePharma (Shanghai, China). Oligonucleotide sequences in this study are shown in Table S1.

For the overexpression plasmids, the full lengths of two different transcripts of porcine *MEG3* gene were synthesized from Tsingke (Beijing, China) and cloned into the pZW1-son plasmid. The coding DNA sequence (CDS) of *SRF* was amplified by PCR and cloned into the pcDNA3.1 plasmid. The major primers used in this study are listed in Table S2. For the dual-luciferase reporter vector plasmid construction, about 300 bp wild-type and mutant sequences of *MEG3* and *SRF*, containing miR-423-5p seed sequence target sites, were inserted into pGL3-Basic vector. Mutant plasmid of *MEG3* was generated by changing the binding site of miR-423-5p from CTGCCCCT to GACGATAG; that of *SRF* was changed from CTGCCCCTCA to GACGGAGTAT.

All the recombinant plasmids were confirmed by sequencing (Sangon Biotech, Shanghai, China).

### 2.4. Cell Transfection

All transient transfections in porcine satellite cells or PK15 cells were performed with Lipofectamine 2000 reagent (Invitrogen, Carlsbad, CA, USA) according to the manufacturer’s instructions.

### 2.5. Nuclear and Cytoplasmic RNA Fractionation

Cells were prepared at both proliferative and differentiated periods. The procedure for separating the nuclear and cytoplasmic RNA fractionation was performed in accordance with previous published reports [40]. RNAs of cytoplasmic and nuclear fractions were extracted with RNAiso reagent (TaKaRa, Otsu, Japan). The locations of *MEG3* (detection with the overlap region primers of MEG3 two transcripts), *GAPDH* (cytoplasmic marker gene) and *Neat1* (a nuclear expression lncRNA) were analyzed by quantitative polymerase chain reaction (qPCR). Primer sequences for qPCR are listed in Table S2.

### 2.6. RNA Extraction, cDNA Synthesis, and Quantitative Polymerase Chain Reaction(qPCR)

Total RNA was extracted from cells using RNAiso reagent (TaKaRa, Otsu, Japan) according to the manufacturer’s instructions. The concentration and quality were measured by a spectrophotometer (Nanodrop 2000, Thermo Fisher Scientific, Wilmington, DE, USA) at 260 and 280 nm. Ratios of absorption (260/280 nm) of all samples ranged from 1.8 to 2.0.

Complementary DNA (cDNA) synthesis for messenger RNA (mRNA) was performed using the PrimeScript RT Reagent Kit with gDNA Eraser (Perfect Real Time) (TaKaRa, Otsu, Japan). For miRNA, stem loop miRNA qRT-PCR primers specific for miR-423-5p and U6 were designed by Vazyme (Nanjing, China) and cDNA was synthesized with miRNA 1st Strand cDNA Synthesis Kit (by stem-loop) (Vazyme, Nanjing, China).

Quantitative polymerase chain reaction (qPCR) for mRNA was carried out on a Bio-Rad CFX96 Real-Time Detection System using TB Green Premix Ex Taq II (Tli RNase H Plus) (TaKaRa, Otsu, Japan). For miRNA, miRNA Universal SYBR qPCR Master Mix (Vazyme, Nanjing, China) was used and analyzed with the 2^−∆∆CT^ method. All primers for qPCR in this study were designed with Primer 5, and primer sequences are listed in Table S2. The 18S ribosomal RNA (*18S rRNA*) and *U6* were used as internal controls.

### 2.7. 5′ and 3′ RACE and Full-Length LncRNA Cloning

To obtain the transcription information and full-length sequences of *MEG3*, SMARTer RACE cDNA Amplification Kit (Clontech, Osaka, Japan) was used for 5′ and 3′ RACE according to the manufacturer’s instructions. The gene-specific primers (GSP) for RACE PCR were designed for 5′ RACE (GSP1) and 3′ RACE (GSP2) amplification. The PCR products were inserted into the pRACE vector and sequenced by Tsingke Biological Technology (Wuhan, China). The gene-specific primers are shown in Table S2.

### 2.8. CCK-8 Assay

Porcine satellite cells were seeded in 96-well plates and transfected with ASO oligo against *MEG3* or *MEG3* overexpression vectors when cell confluence was 40–50%. Then, cell proliferation was monitored with the CCK-8 Cell Counting Kit (Vazyme, Nanjing, China) following the manufacturer’s protocol. And the absorbance at 450 nm was measured in the spectrophotometer after being transfected for 12, 24, 36, and 48 h.

### 2.9. 5-Ethynyl-20-deoxyuridine (EdU) Assay

Porcine satellite cells were transferred to culture medium with 50 μM EdU (RiboBio, Guangzhou, China) for 2 h at 37 °C after 36 h transfection. Afterwards, cells were fixed in 4% paraformaldehyde for 15 min at room temperature (RT), and then permeabilized with 0.3% Triton X-100 for 10 min. To block unspecific binding, cells were incubated in the blocking buffer (PBS containing 3% bovine serum albumin, 0.3% Triton X-100) for 1 h at RT. Then cells were incubated with a solution containing 10 mM EdU in dark for 30 min. The nuclei were stained with 10 µg/mL 4, 6-diamidino-2-phenylindole (DAPI, Invitrogen, Carlsbad, CA, USA) solution in dark for 10 min. Leica SP8 confocal microscope was used to capture three randomly selected fields to visualize the number of EdU-stained cells.

### 2.10. Flow Cytometry Analysis

For flow cytometry analysis of cell cycle, the Cell Cycle Detection Kit (Keygen, Nanjing, China) were used in line with the manufacturer’s instructions. Briefly, cells were harvested and fixed in 70% ethanol overnight at 4 °C after 36 h transfection. Then cells were rinsed with PBS and centrifuged at 2500 rpm for 5 min. Subsequently, cells were stained with prepared propidium iodide (PI) solution, containing RNase A and PI at a volume ratio of 1:9, and then incubated in dark for 30 min at RT. Flow cytometry analysis was performed on Beckman Coulter FC500 Cytometer (Beckman Coulter, Miami, FL, USA) and data were processed by ModFit software (Verity Software House, Topsham, ME, USA).

### 2.11. Transcriptome Sequencing and Differential Expression Analysis

To further study the involvement of *MEG3* in porcine myogenesis, we thoroughly analyzed RNA-seq data from *MEG3* knockdown and control groups in porcine satellite cells differentiated for 30 and 40 h. In total, 3 μg of RNA for each sample was used to construct sequencing libraries. The libraries were sequenced on the Illumina HiSeq X-ten platform and 150 bp paired-end reads were generated. Then, we used FastQC software (Nanjing Agricultural University, Nanjing, China) to evaluate the quality of obtained sequence data and used Trimmomatic tool (version 0.3.2, Nanjing Agricultural University, Nanjing, China) to trim. Next, HISAT2 (version 2.0.1, Iowa State University, Ames, IA, USA) was used to obtain the qualified and clean reads mapped to the pig reference genome (Sus scrofa 11.1) and StringTie (version 1.3.4, Johns Hopkins University, Baltimore, MD, USA) was used to assemble the mapped reads with default parameters. HTSeq-count (version 0.9.1, European Molecular Biology Laboratory, Heidelberg, BW, Germany) was used to count reads mapped to the genome and the annotation file. Subsequently, differentially expressed genes were identified utilizing the R packages DESeq2 (Tsinghua University, Beijing, China). A transcript will be considered as differentially expressed between two groups if the absolute value of log2 (fold-change) > 1, *p*-value < 0.05 and false discovery rate (FDR) < 0.05. In order to query each protein-coding gene and understand their functions, we performed gene ontology (GO) and Kyoto Encyclopedia of Genes and Genomes (KEGG) pathway enrichment analysis by running queries for each protein-coding gene against the DAVID database. GO terms or KEGG pathways with corrected *p*-value < 0.05 were considered to be enriched clusters. Because of the limitation of genes annotation in *Sus scrofa*, all genes were converted into human homologous genes using BIOMART from Ensembl.

### 2.12. Dual-Luciferase Reporter Assay

When cell confluence reached about 80%, the wild-type and mutant dual-luciferase reporter vectors of *MEG3* or *SRF* were separately co-transfected into PK15 cells with pRL-TK normalizing reporter plasmid and miR-423-5p mimic. For the interaction among *MEG3*, miR-423-5p, and *SRF*, wild-type dual-luciferase reporter vectors of *SRF*, miR-423-5p mimic, and different doses of pcDNA3.1-*MEG3* plasmids (0, 1, and 2 μg) were co-transfected into PK15 cells. After incubation for 48 h, the cells were harvested. The firefly and renilla luciferase activities were measured using Dual-Luciferase^®^ Reporter Assay System (Promega, Fitchburg, WI, USA).

### 2.13. RNA Immunoprecipitation Assay MEG3

To identify specific RNA molecules associated with Argonaute2 (*Ago2*), a key effector of small RNA mediated gene silencing [41], we performed RNA immunoprecipitation (RIP) assay with EZ-Magna RIP Kit (Millipore, Billerica, MA, USA) according to the manufacturer’s protocol. Briefly, porcine satellite cells, differentiated for 48 h, were collected and lysed in RIP lysis buffer. Then, cell lysates were incubated with A/G magnetic beads conjugated with anti-Ago2 antibody (Boster, Wuhan, China). Then, the immunoprecipitated RNA was isolated and qPCR was performed to detect the abundance of *MEG3*, *SRF*, and miR-423-5p; *18S rRNA* and *U6* were used as internal controls. Primer sequences for qPCR are listed in Table S2.

### 2.14. Immunofluorescence Staining

Cells were fixed in 4% paraformaldehyde for 15 min after 48 h transfection and then permeabilized in 0.3% Triton X-100 for 10 min. Subsequently, cells were blocked with blocking solution (3% BSA, 0.3% Triton X-100, 10% FBS complemented with PBS) for 2 h. Then, immunofluorescence staining was performed using anti-MyHC (Millipore, Billerica, MA, USA; 1:1000) or anti-MyoG (Abcam, Cambridge, MA, USA; 1:500) overnight at 4 °C. After that, cells were stained with Alexa 594-labeled anti-mouse antibody (Antgene, Wuhan, China; 1:200) for 1 h. The cell nuclei were visualized using DAPI (Invitrogen, Carlsbad, CA, USA) solution in darkness for 10 min. Images from three randomly selected fields were obtained with Leica SP8 confocal microscope and processed with Image J software (version 1.48, National Institutes of Health, Bethesda, MD, USA).

### 2.15. Western Blot

Total protein was extracted from porcine satellite cells and lysed in RIPA lysis buffer with 1% PMSF. The protein concentration was detected with Pierce BCA Protein Assay Reagent (Thermo-Fisher, Waltham, MA, USA). Then, we performed immunoblotting with various antibodies according to standard procedures. The primary antibodies were diluted as follows: MyoG (Abcam, Cambridge, MA, USA) 1:1000, MyoD (Abclonal, Wuhan, China) 1:1000, MyHC (Millipore, Billerica, MA, USA) 1:3000, and β-tubulin (Servicebio, Wuhan, China) 1:2000. The horseradish peroxidase (HRP)-conjugated secondary antibodies (1:4000) (anti-rabbit IgG, or anti-mouse IgG; Servicebio, Wuhan, China) were used to detect protein expression.

### 2.16. Statistical Analysis

Generally, results are presented as the means ± standard error of the mean (SEM). Statistical comparison between two different groups were assessed by two-tailed Student’s *t*-test. The *p* value < 0.05 was considered to be statistically significant.

## 3. Results

### 3.1. Expression of MEG3 LncRNA

**Figure 1:**
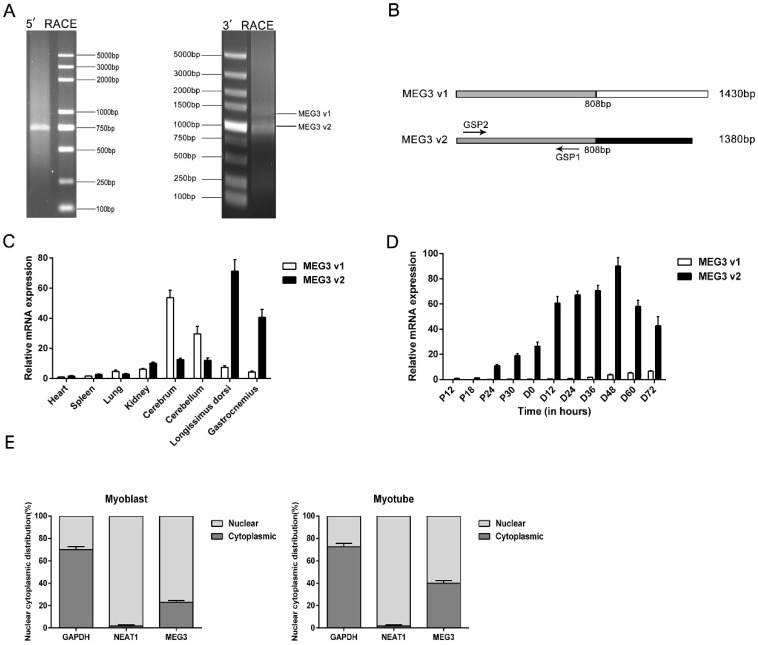
Characterization of the lncRNA *MEG3* gene. (**A**) Results of 5′ rapid amplification of cDNA ends (RACE) and 3′ RACE of lncRNA *MEG3* in porcine skeletal muscle. The arrows represent the two transcripts variants of *MEG3*. (**B**) Structure of two transcript variants (*MEG3* v1 and *MEG3* v2) of the porcine *MEG3* gene. The grey shades mean that the two transcripts share the same front parts. GSP1 and GSP2 are the gene-specific primers for 5′ and 3′ RACE of *MEG3*. (**C**) Expression levels of the two *MEG3* variants in heart, spleen, lung, kidney, cerebrum, cerebellum, longissimus dorsi and gastrocnemius. (**D**) Expression levels of the two *MEG3* variants during porcine myoblast proliferation and differentiation. (**E**) The distribution of *MEG3* core variant transcript (*MEG3* v2) in the cytoplasm and nuclei of porcine proliferous myoblast and myotube differentiated for 48 h. *NEAT1* is a known nuclear lncRNA, and *GAPDH* is a cytoplasmic-enriched gene. The relative RNA levels were normalized to those of the control *18S rRNA*. Error bars represent mean ± SEM of three biological replicates. Statistical significance of differences was assessed by Student’s *t*-test. NC, negative control.

To investigate the regulatory mechanism in pigs, we first evaluated the full-length cDNA of *MEG3* in porcine satellite cells using 5′ and 3′ RACE (Figure 1A). We designed gene-specific primers (GSP) for RACE and identified two polyadenylated *MEG3* transcripts: *MEG3* variant 1 (*MEG3* v1), 1430 nt in length, and *MEG3* variant 2 (*MEG3* v2), 1380 nt in length. Sequence analysis showed that these two different variants shared the same 508-nt front part (Figure 1B). Interestingly, *MEG3* showed remarkably higher mRNA expression levels in brain and muscle tissues, such as longissimus dorsi and gastrocnemius muscle, than other multiple tissues (Figure 1C). Notably, the expression of *MEG3* v2 showed a substantial dominance in porcine muscle tissues compared with *MEG3* v1 (Figure 1C). We collected porcine satellite cells at the proliferation stage every 6 h and at the differentiation stage every 12 h to distinguish the expression levels of these two *MEG3* variants in different periods of myogenesis. Overall, two variants of *MEG3* had similar expression trends. Both variants were upregulated in the proliferation and early stage of differentiation and reached their peaks in porcine satellite cells differentiated for 48 h, but they gradually decreased afterward (Figure 1D), suggesting that *MEG3* could be a promyogenic factor during the early differentiation in porcine satellite cells. Obviously, *MEG3* v2 was the most abundant in porcine satellite cells with dominant expression level compared with that of *MEG3* v1 (Figure 1D). Therefore, *MEG3* v2 was the core transcript in porcine satellite cells. Moreover, nuclear–cytoplasmic RNA fractionation experiments demonstrated that *MEG3* was mainly located in the nuclear compartment of proliferating myoblasts (76.86%) and differentiated myotubes (59.99%). Interestingly, its proportion in the cytoplasm had increased in myotubes (in a range from 23.14% to 40.01%) (Figure 1E).

### 3.2. MEG3 Inhibits Myoblast Proliferation

**Figure 2:**
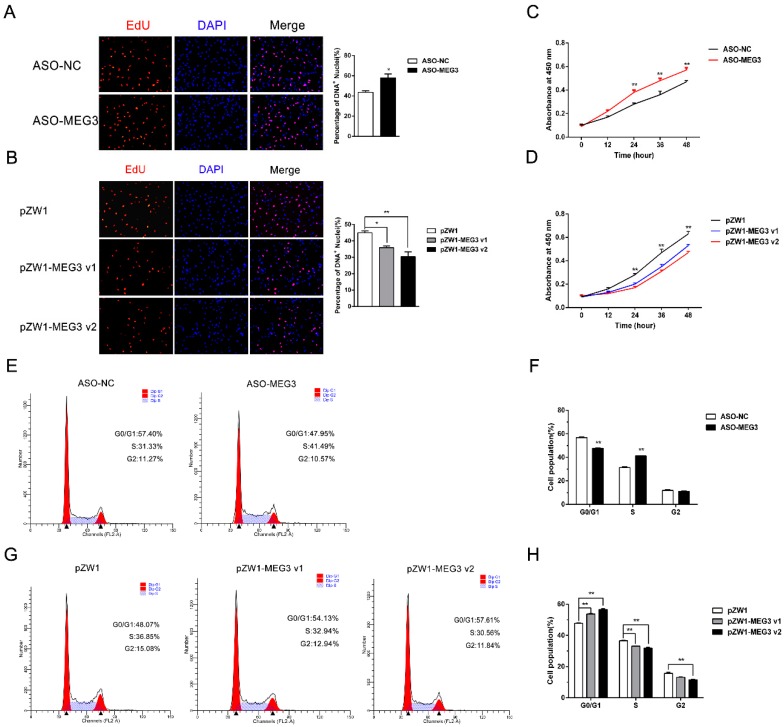
*MEG3* inhibits myoblast proliferation. (**A**,**B**) EdU staining assays after *MEG3* knockdown (**A**) or overexpression of the two *MEG3* variants (**B**) showing the EdU incorporation in proliferous porcine satellite cells. The S phase of mitosis cells were stained with EdU. The nuclei were stained with DAPI. Scale bar: 50 μm. (**C**,**D**) CCK-8 cell proliferation assays of porcine satellite cells suggested that *MEG3* knockdown significantly promoted myoblast proliferation after transfection with antisense oligonucleotide (ASO)–*MEG3* for 24, 36, and 48 h, compared with the negative control groups (**C**), while overexpression of *MEG3* v1 or *MEG3* v2 inhibited myoblast proliferation (**D**). (**E**,**G**) Flow cytometry analysis after *MEG3* knockdown (**E**) or overexpression of the two *MEG3* variants’ overexpression plasmids for 36 h (**G**). (**F**,**H**) Histogram bar graph summarizing the flow cytometry analysis showing the number of cells in each cell cycle phase. Statistical results showing that *MEG3* knockdown significantly increased the percentage of S-phase cells (**F**), while *MEG3* overexpression significantly decreased the percentage of S-phase cells (**H**). Error bars represent mean ± SEM of three biological replicates. Statistical significance of differences was assessed by Student’s *t*-test. * *p* < 0.05, ** *p* < 0.01. NC, negative control.

*MEG3* expression levels were upregulated during the myoblast proliferation phase, indicating that *MEG3* could be involved in the regulation of myoblast proliferation. In functional deficit and acquisition experiments, optimized phosphorothioate-modified antisense oligodeoxynucleotide (ASO) against the overlapping region sequence of the two *MEG3* variants and pZW1-*MEG3* v1 or pZW1-*MEG3* v2 plasmid were respectively transfected into porcine satellite cells. In the 5-ethynyl-2′-deoxyuridine (EdU) staining assays, the interference of *MEG3* with ASO showed higher mitotic activity with an increase in EdU incorporation (Figure 2A). On the contrary, the overexpression of *MEG3* v1 (*p* < 0.05) or *MEG3* v2 (*p* < 0.01) showed lower mitotic activity with a decrease in EdU positivity (Figure 2B). The CCK-8 assay showed that the *MEG3* knockdown for 24, 36, or 48 h could dramatically accelerate cellular proliferation (Figure 2C). Inversely, the overexpression of *MEG3* v1 or *MEG3* v2 substantially suppressed the proliferative ability of porcine satellite cells compared with the negative control (Figure 2D). The propidium iodide flow cytometry assays indicated a considerable reduction of cell quantity in the G0/G1 phase and a remarkable increase of cell quantity in the S phase after *MEG3* knockdown (Figure 2E,F). Conversely, the overexpression of *MEG3* v1 or *MEG3* v2 showed an opposite effect (Figure 2G,H). These findings validated that *MEG3* could inhibit the proliferation of porcine satellite cells.

### 3.3. MEG3 Promotes Myoblast Differentiation

**Figure 3:**
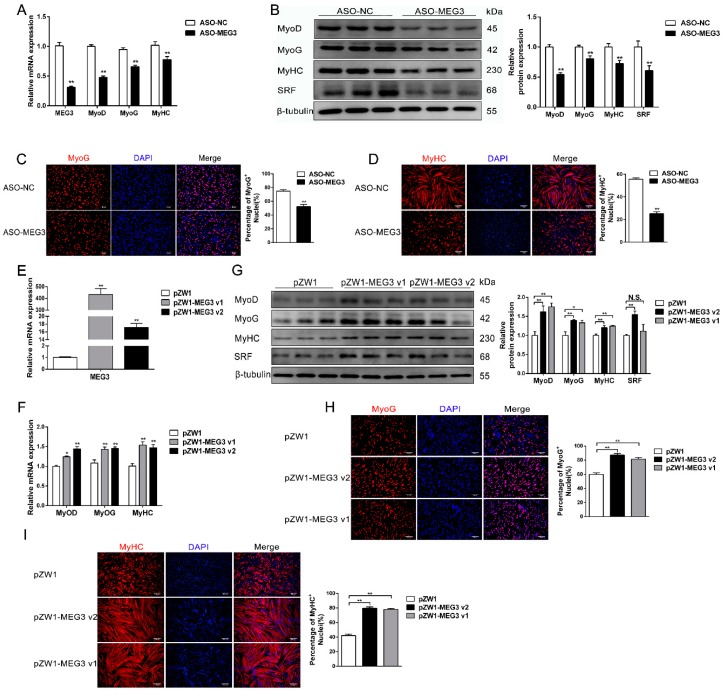
*MEG3* promotes myogenic differentiation of porcine satellite cells. (**A**,**B**) The real-time PCR (**A**) and Western blot analysis (**B**) showing that *MEG3* knockdown significantly decreases expression levels of myogenic marker genes (*MyoD*, *MyoG*, *MyHC*) and *SRF*. (**C**,**D**) Immunofluorescence staining of *MyoG* (**C**) and *MyHC* (**D**) in porcine satellite cells after transfection with ASO–*MEG3* or ASO–NC showing that *MEG3* knockdown significantly reduces the positively stained cells of *MyoG* and *MyHC*. (**E**) The detection of overexpression efficiency of *MEG3* v1 and *MEG3* v2. (**F**–**I**) qPCR (**F**), Western blot (**G**), and immunofluorescence staining of *MyoG* (**H**) and *MyHC* (**I**) results showing that *MEG3* overexpression promotes satellite cell differentiation. Scale bar of (**C**,**H**): 50 μm. Scale bar of (**D**,**I**): 100 μm. Porcine satellite cells were harvested after transfection with *MEG3* ASO or overexpression plasmid and differentiation for 48 h. The relative mRNA levels were normalized to those of the control *18S rRNA*. The relative protein levels were normalized to those of the control *β-tubulin*. Error bars represent mean ± SEM of three biological replicates. Statistical significance of differences was assessed by Student’s *t*-test. * *p* < 0.05, ** *p* < 0.01. N.S. means that there was no significant difference. NC, negative control. qPCR, quantitative polymerase chain reaction.

The above results demonstrated that *MEG3* is extremely important for myoblasts to be able to withdraw from the cell cycle, a crucial step in myoblast differentiation. In addition, the expression profile of *MEG3* prompted its association with myoblast differentiation. We used qPCR, Western blot, and immunofluorescence staining to test the changes of three established myogenic marker genes (*MyoD*, *MyoG*, and myosin heavy chain (*MyHC*)). qPCR results showed that *MEG3* was successfully knocked down in the ASO–*MEG3* group myotube differentiated for 48 h (Figure 3A). Meanwhile, the mRNA and protein levels of *MyoD*, *MyoG*, and *MyHC* were remarkably downregulated after *MEG3* knockdown compared with the control group (Figure 3A,B). Consistently, immunofluorescence staining of *MyoG* and *MyHC* showed that *MEG3* knockdown notably reduced the proportion of *MyoG*^+^ and *MyHC*^+^ cells (Figure 3C,D). To further confirm the above observation, we successfully overexpressed *MEG3* v1 and *MEG3* v2 in porcine satellite cells in the differentiation phase (Figure 3E). As expected, the mRNA and protein levels of *MyoD*, *MyoG*, and *MyHC* substantially increased after *MEG3* v1 and *MEG3* v2 overexpression in myotube (Figure 3F,G). Likewise, *MEG3* overexpression showed that more cells proceeded with differentiation and myotube formation than the control group in the immunofluorescence staining of *MyoG* and *MyHC* (Figure 3H,I). Taken together, our results confirmed that *MEG3* is required for myoblast differentiation.

### 3.4. Gene Expression Profile of MEG3 Knockdown in Porcine Satellite Cells

**Figure 4:**
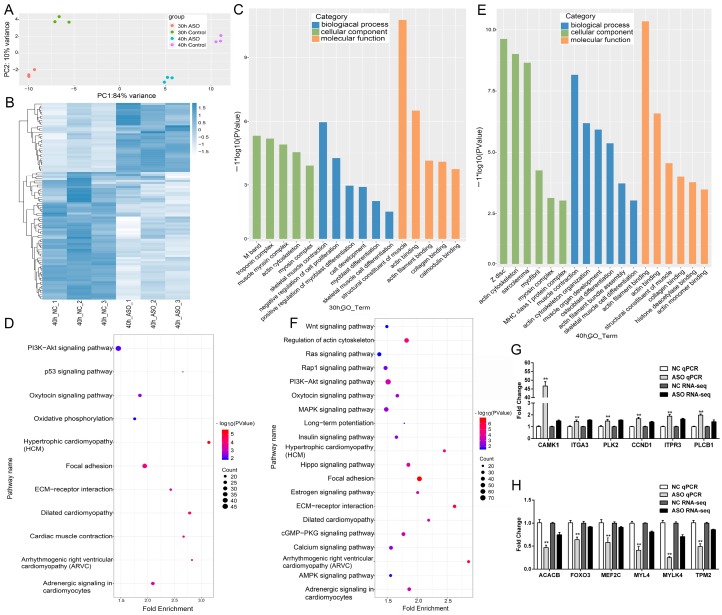
Effect of *MEG3* downregulation on myogenesis transcriptome. (**A**) PCA analysis of four datasets (30 hNC, 30 hASO and 40 hNC, 40 hASO groups) for *MEG3* knockdown. (**B**) Hierarchical clustering of differentially expressed genes after transfection of porcine satellite cells with ASO–*MEG3* or ASO–NC and differentiation for 40 h. The color-ratio bar at the right indicates intensity of gene upregulation (blue) and downregulation (white). (**C**–**F**) GO enrichment and KEGG pathway scatterplot analysis for differentially expressed genes for cells differentiated for 30 (**C**,**E**) and 40 h (**D**,**F**). (**G**,**H**) Validation of differentially expressed genes involved in muscle development by qPCR. The *y*-axis indicates the fold change of RNA-Seq and qPCR. The relative mRNA levels were normalized to those of the control *18S rRNA*. Error bars represent mean ± SEM of three biological replicates. Statistical significance of differences was assessed by Student’s *t*-test. ** *p* < 0.01. NC, negative control. ASO, antisense oligonucleotide. PCA, Principal component analysis. GO, Gene ontology. KEGG, Kyoto Encyclopedia of Genes and Genomes. qPCR, quantitative polymerase chain reaction.

To further study the involvement of *MEG3* in skeletal muscle development, we thoroughly analyzed RNA-seq data from *MEG3* knockdown and control groups in porcine satellite cells differentiated for 30 and 40 h and identified differentially expressed genes between the two groups. In total, we obtained over 40 million raw reads from each library. Then we removed low-quality sequences, and the clean reads mapped more than 95% of the raw data. Next, we aligned all clean reads to the porcine Sscrofa11.1 reference genome and found that more than 70% clean reads could be uniquely mapped to the genome (Table S3). The principal component analysis (PCA) score plots showed that datasets from four groups (30 hNC, 30 hASO and 40 hNC, 40 hASO groups for *MEG3* knockdown) were clustered separately (Figure 4A). Hierarchical clustering was performed for differential expressions of protein-coding genes and obtained a global overview of gene expression profile among 40 hNC group and 40 hNC ASO group for *MEG3* knockdown (Figure 4B). Finally, the differentially expressed genes were used to perform gene ontology (GO) enrichment analysis and Kyoto Encyclopedia of Genes and Genomes (KEGG) pathway analysis. In detail, we identified 273 and 207 differentially expressed protein-coding genes from *MEG3* knockdown for the 30 and 40 h groups, respectively, compared with the corresponding negative control groups. At 30 h comparison groups, GO enrichment analysis of these differentially expressed genes indicated that they were mainly associated with muscle-related processes, including muscle myosin complex, skeletal muscle contraction, and positive regulation of myoblast differentiation, skeletal muscle cell differentiation, and structural constituent of muscle (Figure 4C). KEGG pathway enrichment analysis was used to explore the biological pathways for differentially expressed protein-coding genes. The results showed that these genes participated in the PI3K–Akt signaling pathway, oxytocin signaling pathway, focal adhesion, and adrenergic signaling in cardiomyocytes (Figure 4D). The GO enrichment analysis at 40 h indicated that the differentially expressed genes were also related to muscle-related processes, such as Z disc, actin cytoskeleton, myofibril, muscle contraction, skeletal muscle cell differentiation, and structural constituent of muscle (Figure 4E). KEGG pathway enrichment analysis showed that these differentially expressed genes were mainly involved in the PI3K–Akt signaling pathway, regulation of actin cytoskeleton, focal adhesion, and MAPK signaling pathway (Figure 4F). Among these differentially expressed genes, *CCND1* [42], *PLCB1* [43], *MEF2C* [44], and *FOXO3* [45,46] have a known function in regulating myogenesis. As a consequence, *MEG3* could specifically have important effects on skeletal muscle development. To validate the reliability of our sequencing results, we randomly selected several differentially expressed genes in 30 and 40 h groups for qPCR to detect their expression levels. The expression levels of *CAMK1*, *ITGA3*, *PLK2*, *CCND1*, *EIF4E*, *ITGA7*, *ITPR3*, and *PLCB1* were upregulated, whereas those of *ACACB*, *FOXO3*, *MEF2C*, *MYL4*, *MYLK4*, and *TPM2* were downregulated (Figure 4G,H).

### 3.5. MEG3 Acts As a ceRNA for miR-423-5p

**Figure 5:**
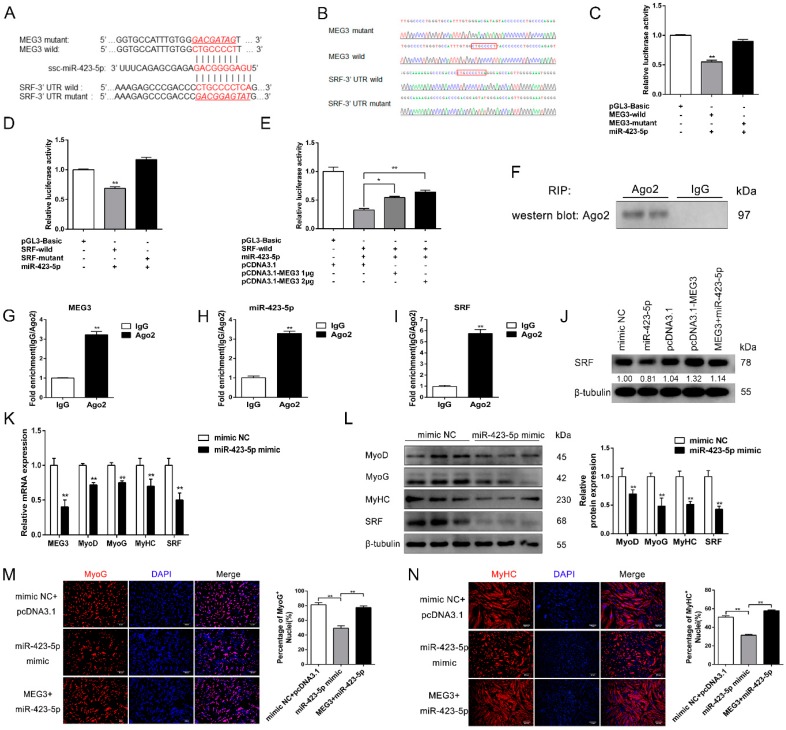
*MEG3* is a target of miR-423-5p during myoblast differentiation. (**A**) The miR-423-5p target sequence on *MEG3* and 3′ UTR of *SRF*. The red letters represent the seed region of miR-423-5p and its target region. The underlined word letters in red show the mutated site. (**B**) The sequencing results of *MEG3* and *SRF*-3′ UTR wild and mutant types. The red boxes represent the mutant bases. (**C**) Luciferase activities assay in PK15 cells were measured after transfection for 48 h with *MEG3* wild or mutant type pGL3-Basic plasmids together with miR-423-5p mimic. (**D**) Luciferase activities assay in PK15 cells were measured after transfection of 48 h with *SRF* wild or mutant type pGL3-Basic plasmid together with miR-423-5p mimic. (**E**) Wild type of *MEG3* fragments rescued the relative luciferase activities of *SRF* wild-type pGL3-Basic plasmid in a dose-dependent manner. (**F**–**I**) RIP assay was conducted with *Ago2* antibody. Protein level was detected by IP-Western Blot (**F**). *MEG3* (**G**), *SRF* (**H**), and miR-423-5p (**I**) were detected by qPCR. (**J**) The protein expression level of *SRF* showing *MEG3* overexpression increases *SRF* expression and relieves the inhibitory effect induced by miR-423-5p on *SRF*. (**K**,**L**) Relative mRNA (**K**) and protein expression level (**L**) changes of MEG3, SRF, and the differentiation marker genes after transfection with miR-423-5p mimic or mimic NC. (**M**,**N**) Immunofluorescence staining of *MyoG* (**M**) and *MyHC* (**N**) in porcine satellite cells differentiated for 48 h after transfection with miR-423-5p mimic, *MEG3*+miR-423-5p mimic, or corresponding NC. Statistical results of the positively stained cells show that miR-423-5p significantly reduces the percentage of *MyoG* and *MyHC*, while the co-transfection with *MEG3* can recover their reduction. Scale bar of (**M**): 50 μm. Scale bar of (**N**): 100 μm. The numbers below the Western blots (**J**) mean the fold change of *SRF* protein quantities related to the mimic NC group. The relative mRNA levels were normalized to those of the control *18S rRNA*. The relative protein levels were normalized to those of the control *β-tubulin*. Error bars represent mean ± SEM of three biological replicates. Statistical significance of differences was assessed by Student’s *t*-test. * *p* < 0.05, ** *p* < 0.01. NC, negative control. UTR, untranslated regions. RIP, RNA immunoprecipitation. qPCR, quantitative polymerase chain reaction.

Given that lncRNAs can regulate multiple biological functions by sponging regulatory miRNAs, the seed regions of miR-423-5p were predicted to be complementary with *MEG3* core transcript *MEG3* v2 (abbreviated as *MEG3*), and another potential target gene (i.e., *SRF*) of miR-423-5p was obtained using TargetScan (http://www.targetscan.org/vert_71/) and RNAhybrid software (https://bibiserv.cebitec.uni-bielefeld.de/rnahybrid/) (Figure 5A). *SRF* is a crucial transcription factor that regulates muscle proliferation and differentiation [47,48], and miR-423-5p is also involved in skeletal muscle development and regeneration [49]. Therefore, we hypothesized that *MEG3* and *SRF* are functional targets of miR-423-5p, that is, *MEG3* modulates *SRF* by competing for miR-423-5p. To determine the binding sites between miR-423-5p and its target gene *MEG3* or *SRF*, we constructed wild-type and mutant versions of *MEG3* and SRF-3′UTR using pGL3-Basic vectors. As shown in Figure 5B, the wild-type and mutant reporter vectors contained miR-423-5p binding sites and mutated recognition sequences, respectively. Different vectors were co-transfected into PK15 cells with miR-423-5p mimic. In a dual luciferase reporter assay, we found that the luciferase activity of wild-type *MEG3* and *SRF* remarkably decreased with the overexpression of miR-423-5p compared with the negative control groups (*p* < 0.01). However, no significant change was observed in the luciferase activity of the mutant group lacking miR-423-5p binding site (Figure 5C,D). Interestingly, *MEG3* could attenuate the decrease of luciferase activity induced by miR-423-5p in a dose-dependent manner (Figure 5E). Consistently, RNA immunoprecipitation (RIP) assay using an antibody against *Ago2* precipitated the *Ago2* protein from our cellular extract of porcine satellite cells differentiated for 48 h. The RIP-Western blot result suggested that *MEG3* had the potential to combine with miRNAs (Figure 5F). The following qPCR results showed that *MEG3* (Figure 5G), *SRF* (Figure 5H), and miR-423-5p (Figure 5I) were significantly enriched in *Ago2* pellet, confirming the interaction among *MEG3*, miR-423-5p and *SRF*. Western blot results indicated that *MEG3* overexpression markedly increased *SRF* expression, while miR-423-5p showed opposite effect on *SRF*. It is worth noting that the inhibitory effect of miR-423-5p on *SRF* could be relieved by co-transfection with *MEG3* overexpression plasmid (Figure 5J). Consistent with the Western blot results in Figure 3B,G, the knockdown or overexpression of *MEG3* could respectively downregulate or upregulate the protein of *SRF* (*p* < 0.01). These findings confirmed the potential of *MEG3* to combine with miRNAs, revealed the connection among *MEG3*, *SRF,* and miR-423-5p, and verified the sponge role of *MEG3* for miR-423-5p.

To further confirm the function of *MEG3* as a ceRNA for miR-423-5p regulating myoblast differentiation, we performed qPCR and Western blot experiments. The results demonstrated that miR-423-5p overexpression significantly inhibited *MEG3*, *SRF*, *MyoD*, *MyoG*, and *MyHC* expressions at the mRNA and protein levels (Figure 5K,L). Meanwhile, the immunofluorescence staining assay of *MyoG* and *MyHC* demonstrated that the number of *MyoG*^+^ and *MyHC*^+^ cells was dramatically reduced after miR-423-5p overexpression (*p* < 0.01); however, transfection with *MEG3* overexpression plasmid could substantially relieve the inhibitory effect on myoblast differentiation (Figure 5M,N). These findings verified that *MEG3* regulates myoblast differentiation via abrogating the role of miR-423-5p.

**Figure 6:**
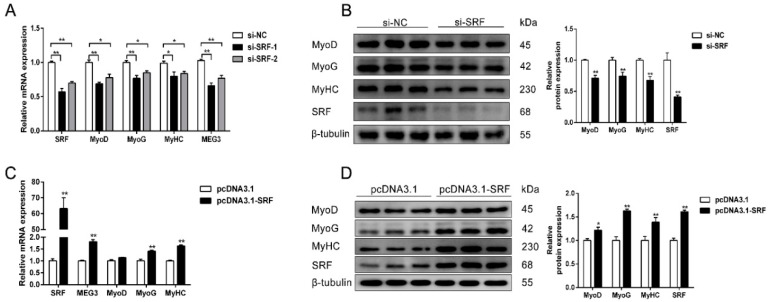
*SRF* promotes the differentiation of porcine satellite cells. (**A**) qPCR results showing that *SRF* knockdown significantly decreases the expression levels of *MEG3*, *MyoD*, *MyoG*, and *MyHC* in in porcine satellite cells differentiated for 48 h. (**B**) Western blot analysis results showing *SRF* knockdown significantly decreases the protein expression of *SRF* and the differentiation marker genes. (**C**,**D**) qPCR (**C**) and Western blot (**D**) results showing *SRF* overexpression significantly increases the expression levels of *MEG3* and promotes porcine satellite cell differentiation. The relative mRNA levels were normalized to those of the control *18S rRNA*. The relative protein levels were normalized to those of the control *β-tubulin*. Error bars represent mean ± SEM of three biological replicates. Statistical significance of differences was assessed by Student’s *t*-test. * *p* < 0.05, ** *p* < 0.01. NC, negative control. qPCR, quantitative real-time polymerase chain reaction.

**Figure 7:**
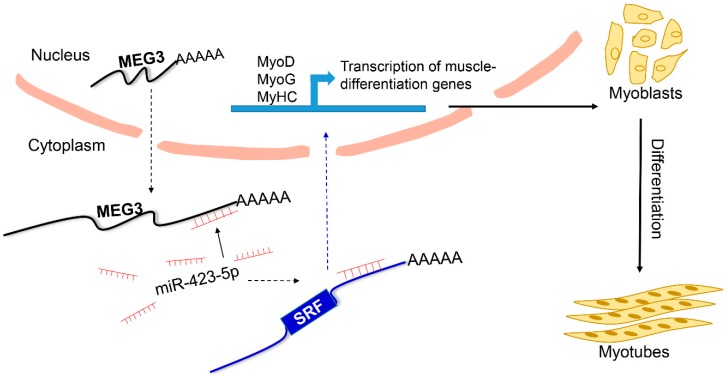
Proposed model of *MEG3* regulatory mechanism in porcine satellite cell differentiation. In this model, *MEG3*, as a ceRNA, promotes porcine satellite cell differentiation by sponging miR-423-5p to relieve the inhibiting effect on *SRF*.

To verify the function of *SRF*, the target gene for miR-423-5p, we transfected the small interfering RNA (siRNA) of *SRF* or *SRF* overexpression plasmid into porcine satellite cells and induced differentiation for 48 h. qPCR results demonstrated that *SRF* knockdown remarkably downregulated *MEG3* mRNA level (Figure 6A). Meanwhile, mRNA and protein expression levels of *MyoD*, *MyoG*, and *MyHC* were considerably decreased, suggesting that si-SRF could inhibit the differentiation of porcine satellite cells (Figure 6A,B). As expected, *SRF* overexpression considerably upregulated the expression of *MEG3* and promoted the differentiation of porcine satellite cells (Figure 6C,D). Collectively, *MEG3* acts as a ceRNA for miR-423-5p to attenuate the inhibitory effect on *SRF*, thereby promoting the differentiation of porcine satellite cells (Figure 7).

## 4. Discussion

In this study, we demonstrated that *MEG3* acted as a key regulator in myogenesis and revealed a novel molecular mechanism by which *MEG3* regulated the miR-423-5p–SRF axis. Our results suggested that *MEG3*, as a ceRNA, promotes the differentiation of porcine satellite cells by sponging miR-423-5p to relieve the inhibiting effect on *SRF* (Figure 7).

Proliferation and differentiation of myoblasts are crucial processes for skeletal muscle development, which determine the quality and quantity of agricultural animal meat production. Elucidating the regulatory mechanisms of myogenesis helps to find therapeutic targets for muscle disease and improve meat traits in animal production. Therefore, understanding the underlying mechanisms of myogenesis is particularly important. Although thousands of lncRNAs have been identified [38,50], a minority of functional lncRNAs, such as *TncRNA* [51], *lncMD* [52], *H19* [52,53,54,55,56], and *lncIRS*1 [57], are involved in mammal myogenesis. *MEG3*, a differentially expressed lncRNA during postnatal skeletal muscle development in pigs, has four crucial polymorphism sites associated with back fat thickness [39]. Li et al. first confirmed the two overlapping fragment isoforms of *MEG3* with respective lengths of 1160 and 1219 bp in Yorkshire and Korean native pigs [27]. Our RACE results confirmed the two variants in pigs and showed that they were 1430 and 1380 bp in full length. Subsequently, the tissue expression profile showed the advantage of their expression and the high abundance of *MEG3* v2 in skeletal muscle tissues, which was consistent with the results of a previous study in various tissues and six developmental stages of longissimus dorsi muscle during porcine postnatal development [58]. In addition, the high expression level of *MEG3* during the early stage of myogenesis in pigs and cattle collectively indicated its important role in myogenesis and muscle development [35]. Our research results demonstrated that *MEG3* knockdown remarkably decreased the expression of myogenic marker genes in mRNA and protein levels; however, the overexpression results of the two transcripts of *MEG3* were opposite. The findings conceivably indicated that *MEG3* could act as an accelerator in porcine myogenic differentiation, which is also consistent with a previous study in cattle [35]. Furthermore, we further assessed differential gene expression after *MEG3* knockdown, with differentiation for 30 and 40 h, and provided global insights into gene functions during myogenesis using an RNA-seq approach. KEGG pathway and GO term enrichment analysis found the involvement in muscle-related processes, such as Z disc, actin cytoskeleton, myofibril, muscle contraction, skeletal muscle cell differentiation, and structural constituent of muscle. As a consequence, it is reasonable to infer that *MEG3* plays an important role in regulating porcine skeletal muscle development.

To reveal the underlying molecular mechanism of *MEG3*, we explored a large number of existing studies. We found that several functional lncRNAs have been characterized, and their function can be affected by multiple mechanisms. Partial lncRNAs, such as the new *lncRNA SYISL* [25], *lncRNA Mata1* [59], and *Linc-YY1* [24], regulate myoblast differentiation and skeletal muscle regeneration by recruiting chromosome modification complexes to the promoters of target genes. Other lncRNAs, such as *Linc-RAM* [40] and *Myoparr*, can recruit transcription factor *MyoD* and RNA-binding protein complex Ddx17/PCAF, respectively, to myogenic marker gene promoters to further promote myogenic differentiation and regeneration [60]. Also, *LncMyoD* acts as a competitive binding regulator to attenuate the binding ability of *IMP2* for its target genes and inhibit myoblast differentiation [22]. Notably, subcellular localization determines the regulatory mechanism. Many cytoplasm-located lncRNAs can act as a ceRNA to sponge miRNA and relieve the inhibitory effect on target genes. Muscle-specific lncRNA, *Linc-MD1*, serves as a molecular sponge of miR-133 and miR-135 to relieve the repression of *MAML1* and *MEF2C* and induce skeletal muscle differentiation [19]. Overexpression of *lncRNA MAR1* can promote myogenic differentiation by effectively weakening the inhibitory effects of miR-487b on *Wnt5a* [61]. *MEG3* is mainly found in the nucleus of porcine satellite cells, and very rarely is located in the cytoplasm [33,62]. However, the proportion of *MEG3* in the cytoplasm was remarkably increased in the myotube in the present study. This result led us to hypothesize that *MEG3* may act as a ceRNA to regulate the differentiation of porcine satellite cells similar to cytoplasm-located lncRNAs. Similarly, miR-9 can regulate the expression of nuclear *lncRNA MALAT1* by directly binding with miRNA recognition elements and in an Argonaute-2-dependent manner in human L428 and U87MG cells [63]. In addition, *MALAT1* is translocated from the nucleus into the cytoplasm during the G2/M cell cycle phase by interacting with heterogenous nuclear RNP C in the cytoplasm [64], where *MALAT1* acts as a ceRNA for miR-133 and modulates *SRF* to promote the differentiation of C2C12 cell line [59]. This may be a reasonable explanation for the increased proportion of *MEG3* in the cytoplasm from primary porcine myoblast to myotube in the present study. Therefore, we further affirmed the molecular sponge role of *MEG3*.

Software prediction analysis revealed that *MEG3* certainly shared the same miRNA recognition sites for miR-423-5p with *SRF*, a crucial transcription factor for myogenesis process. Previous research showed functional *SRF* is required for the differentiation of C2C12 cells and the regulation of *MyoD* expression [47]. Deletion of *SRF* severely suppresses the muscle formation of muscle progenitors in mammalian embryonic development process [48], blocks cell fusion, and inhibits the synthesis of *MyoD*, *MyoG*, and *MyHC*, exerting severe muscle atrophy [47,65]. Similarly, *SRF* mutant mice died from severe skeletal muscle myopathy characterized by a deficiency in muscle growth during the perinatal period by inhibiting the recruitment of myocardin-related transcription factors [48,66,67]. Our observations in *SRF* siRNA and overexpression group provided evidence on the role of *SRF* in myogenesis. In addition, we found that *SRF* had conservative complementary sites with miR-423-5p, which is a potential regulator of myogenesis and plays a negative role during myoblast proliferation and differentiation by targeting the suppressor of fused homolog [49]. Because the Ago-RIP method described in Werfel et al. had been performed to seek ceRNA for a specific miRNA [68], we carried out an RNA immunoprecipitation assay using antibody against *Ago2*. The RIP-Western blot result showed the potential of *MEG3* to combine with miRNAs. The enrichment of *MEG3*, *SRF* and miR-423-5p in *Ago2* pellet revealed the connection among them. Subsequently, luciferase activity assays verified that *MEG3* acted as a molecular sponge to adsorb miR-423-5p. *MEG3* overexpression could effectively recover the reduced luciferase activity of wild-type *MEG3* and *SRF* induced by miR-423-5p. Consistently, the inhibitory effect of miR-423-5p on *SRF* and myoblast differentiation could be abolished by *MEG3* overexpression. These findings verified the novel functional mechanism that *MEG3* acts as a ceRNA to sponge miR-423-5p, which weakens the suppression on *SRF*, thereby promoting the differentiation of porcine satellite cells. In addition, the fact that *MEG3* was detected mainly in the nucleus implies that it can regulate myogenesis though other mechanisms, which remain to be further explored.

In conclusion, *MEG3* is a vital regulator that inhibits myoblast proliferation and promotes myoblast differentiation in porcine satellite cells. Our findings suggested the novel functional mechanism of *MEG3*, which acts as a molecular sponge of miR-423-5p to upregulate the target gene *SRF* expression level during the differentiation of porcine satellite cells. Our research provides new insights into the molecular mechanisms of *MEG3* in porcine myogenesis and contributes to a better understanding of molecular regulation of lncRNA in multiple pathways.

## Supplementary Materials

The following are available online at https://www.mdpi.com/2073-4409/9/2/449/s1, Supplementary Table S1: Oligonucleotide sequences in this study, Supplementary Table S2: Information of Primers, Supplementary Table S3: Summary of RNA-seq data.
