# Supplementary material for: MEG3 Promotes Differentiation of Porcine Satellite Cells by Sponging miR-423-5p to Relieve Inhibiting Effect on SRF
Source: Cells. 2020 Feb 15;9(2):449. doi: 10.3390/cells9020449 (PMC7072828; doi:10.3390/cells9020449)
Supplement: Supplementary file 1 [file cells-09-00449-s001.pdf]

Table S1. Oligonucleotide sequences in this study.

Fragment name	Sequence(5'-3')
ASO-MEG3	GCCAGTGAGATGCTGGTTGT
miR-423-5p mimic	UGAGGGGCAGAGAGCGAGACUUU
si-SRF-1	GCAAGAGGAAGACGGGCAUTT
si-SRF-2	CCCGCAAACUGCAGCCCAUTT

Table S2. Information of Primers

Gene or Primer name	Primer	Primer sequence(5'-3')
18S rRNA	Forward primer	CCCACGGAATCGAGAAAGAG
	Reverse primer	TTGACGGAAGGGCACCA
MEG3 v1	Forward primer	TCGCTGGCTTCCTTGCTC
	Reverse primer	GGTCCGTGGTGACTCTCCC
MEG3 v2	Forward primer	GTAGGGCTCCACCCAGACG
	Reverse primer	GACGCAGTAACAACGCAGG
MEG3 overlap(OL)	Forward primer	GATCCCACCAGCCTACGAA
	Reverse primer	GACCAATGCCCCCTCACTA
GAPDH	Forward primer	CGTCCCTGAGACACGATGGT
	Reverse primer	GCCTTGACTGTGCCGTGGAAC
Neat1	Forward primer	GTCGATGCCCTGAACATG
	Reverse primer	GGTTAGCACGGAACCTACA
MEG3 (MEG3 v2)	Forward primer	GTAGGGCTCCACCCAGACG
	Reverse primer	GACGCAGTAACAACGCAGG
MyoD	Forward primer	GGCTGCCCAAGGTGGAAATC
	Reverse primer	TGCGTCTGAGTCACCGCTGTAG
MyoG	Forward primer	ATGAGACATCCCCCTACTTCTACCA
	Reverse primer	GTCCCCAGCCCCTTATCTTCC
MyHC	Forward primer	GTTCAGAGAAAGGCATCCCAAA
	Reverse primer	GAGAGTGACCGACACCACAAGTG
CAMK1	Forward primer	CGCCAAAGACTTCATCCGACA
	Reverse primer	TTCTTCTTGATCTGCTCGCTCA
ITGA3	Forward primer	GCATCCCACGAACATCACC
	Reverse primer	ATAAACGGCACCCCCTACC
PLK2	Forward primer	GAAGACCTCGGCTCTACCTCC
	Reverse primer	TTGTAGATATCCTGTCCTCGTTG
CCND1	Forward primer	CCATGAACTACCTGGACCGCTT
	Reverse primer	TGGTCTCCTTCATCTTCGAGGC
ITPR3	Forward primer	GCACAACAACCGCAAGC
	Reverse primer	TGGACACACAAAGGTCAGAGA
PLCB1	Forward primer	TGAGAAGGAGGGCAGCTTTG
	Reverse primer	TTGGGTCATTTCGGCATCCA

ACACB	Forward primer	GACAAGAAGCAAGAGCACATCA
	Reverse primer	GGAACAAGCCAGCACCAGA
FOXO1	Forward primer	TGGTGGCGCAAATGAGTA
	Reverse primer	CAGCAGCACAGTGTTTGGAC
MEF2C	Forward primer	ACAGGTGGTCTGATGGGTG
	Reverse primer	ATGGGGGGAGGAGATTTT
MYL4	Forward primer	GCCTTTTCGCTGTTTGACC
	Reverse primer	TGACTGTGCCGTTGCTCTC
MYLK4	Forward primer	GTGTGCCTTCTCTCGTCTCC
	Reverse primer	CTGGGTGTCCTCAAGAGCAG
TPM2	Forward primer	AGCCCAAGCGGACAAGTATT
	Reverse primer	CCAAGGTCTCTTCCAGGTCATC
ssc-miR-423-5p	Forward primer	AGGGGCAGAGAGCGAGACTTT
	Reverse primer	AGTGCAGGGTCCGAGGTATT
U6	Forward primer	CGCTTCGGCAGCACATATAC
	Reverse primer	TTCACGAATTTGCGTGTCAT
SRF	Forward primer	CCTCTCACTCCAGCCAAAGAA
	Reverse primer	GCCCCACCATCAACAGAAA
MEG3-GSP1	5' RACE primer	ATGAGACGGTGAGACACGGGAGCGG
MEG3-GSP2	3' RACE primer	GACGACCAAGGAGGAGGACGACCAG
Sl-ssc-miR-423-5p	Reverse transcription primer	GTCGTATCCAGTGCAGGGTCCGAG GTATTCGCACTGGATACGACAAAGTC
SRF CDS	Forward primer	GCAGCCTGAACCGGACCCC
	Reverse primer	TGAAGCTGAACCGCCGAGAC

Table S3. Summary of RNA-seq data.

Subjects	Accession number	Raw reads	Clean reads	Mapping ratio (%)	Uniquely mapping ratio (%)
30h_ASO_1	SRR10998733	45444516	44875755	43224327(96.32%)	32870857 (73.25%)
30h_ASO_2	SRR10998732	44374389	43755268	42070690(96.15%)	32099543 (73.36%)
30h_ASO_3	SRR10998729	48383177	47957045	45813365(95.53%)	33910157 (70.71%)
30h_NC_1	SRR10998728	40440484	40032553	38603390(96.43%)	29396372 (73.43%)
30h_NC_2	SRR10998727	54040518	53379847	51431482(96.35%)	39264664 (73.56%)
30h_NC_3	SRR10998726	50476331	49825092	47832088(96%)	36237211 (72.73%)
40h_ASO_1	SRR10998725	45760689	45381467	43706890(96.31%)	32287507 (71.15%)
40h_ASO_2	SRR10998724	46403816	46000772	44294143(96.29%)	32948294 (71.63%)
40h_ASO_3	SRR10998723	44359575	43919793	42400168(96.54%)	32043245 (72.96%)
40h_NC_1	SRR10998722	42273131	41833659	40361114(96.48%)	30813983 (73.66%)
40h_NC_2	SRR10998731	46564797	46082779	44400757(96.35%)	32861771 (71.31%)
40h_NC_3	SRR10998730	43424722	42954258	41352064(96.27%)	31319977 (72.91%)
